# Aqueous Electrochemical Direct Air Capture Using Alizarin Red S

**DOI:** 10.1002/cssc.202401315

**Published:** 2024-10-30

**Authors:** Samuel R. Wenger, Deanna M. D'Alessandro

**Affiliations:** ^1^ School of Chemical and Biomolecular Engineering Faculty of Engineering The University of Sydney Darlington, NSW 2008 Australia; ^2^ School of Chemistry Faculty of Science The University of Sydney Camperdown, NSW 2006 Australia

**Keywords:** Carbon dioxide removal, Direct Air Capture, Electrochemistry, Green chemistry, Redox flow

## Abstract

Direct Air Capture (DAC) is an emerging form of atmospheric carbon dioxide removal. Conventional DAC sorbents utilize swings in temperature and/or pressure, which are energy intensive and hinders large‐scale deployment. In this work, we demonstrate a green, aqueous electrochemical DAC system that employs Alizarin Red S (ARS) as an electroactive capturing agent. The system has an estimated minimum theoretical energy requirement of 24.6 kJe/mole of CO_2_, demonstrated reversible electrochemical behavior over 100 cycles and 205 hours, and maintained an average coulombic efficiency of 100 % with an average capacity retention of 99.8 %. With a techno‐economic analysis, we highlight the impact of current density and electrode surface area on levelized costs, and we describe a path to lower the cost of DAC below US$500 per tonne of CO_2_.

## Graphical abstract

This journal article describes the development and characterization of a green, aqueous electrochemical Direct Air Capture process. It also describes the engineering of a 3D printed redox flow cell. Additionally, it includes a techno‐economic analysis with insights into lowering the process’ costs.

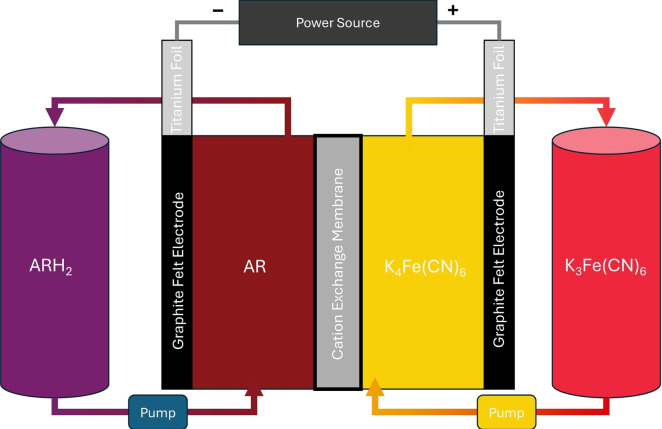

## Introduction

In the design of Direct Air Capture (DAC) systems, energy consumption, cyclability, and raw material costs are of great concern because they have a significant impact on the levelized cost of CO_2_ removal.^[1]^ Thus far, a variety of processes reported in the literature have employed metal hydroxides,^[2]^ amines,[^3^, ^4^] zeolites,^[5]^ metal‐organic frameworks,[^6^, ^7^] and sedimentary rocks^[8]^ as active sorbent materials for DAC. The reported research on these materials has characteristically involved swings in temperature and pressure to reversibly capture and release CO_2_.[^1^, ^9^] More specifically, sorbent materials capture CO_2_ from the air under ambient temperature and pressure conditions, and then elevated temperatures and/or reduced pressures either trigger desorption if the molecules are relatively weakly bound via physisorption, or bond breaking in the case of chemisorption.

Most DAC systems suffer from mass transport limitations due to the dilute nature of CO_2_ in the air (0.04 % by volume). Therefore, to increase mass transport and the rate of capture, sorbent materials are typically coated onto inactive substrates to enhance the surface area of the active sorbent.[^1^, ^2^, ^8^] In this regard, temperature/pressure swing systems utilize significant quantities of dispersants, coating materials, and substrates – all of which do not actively capture CO_2_. When such DAC systems perform desorption, inactive materials absorb heat, which results in wasted energy and a sub‐optimal process.

To address these inefficiencies, electrochemical DAC systems utilize swings in electrical potential to oscillate electroactive molecules between their redox states.^[10]^ These electrochemical processes can be relatively more energy efficient because energy is not lost in the translation of electricity into heat, and energy is minimally applied to inactive components. Inefficiencies in these systems manifest as overpotential, which can be addressed by optimizing solvent systems and improving electrochemical cells in order to minimize wasted energy.[^11^, ^12^]

**Scheme 1:**
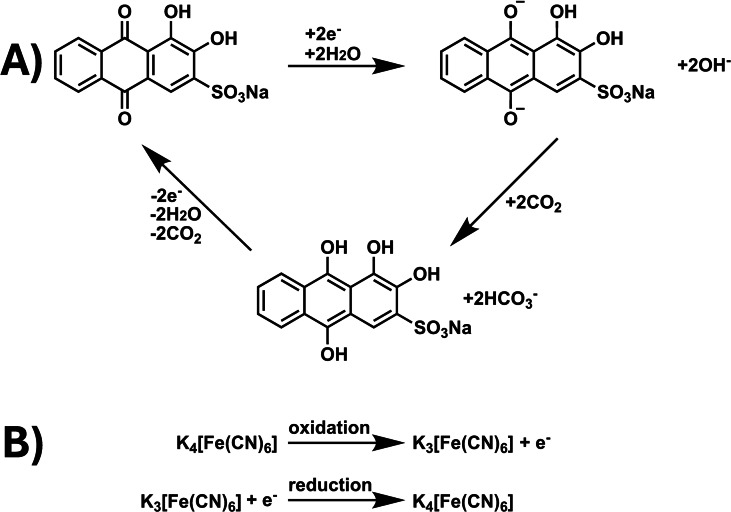
The proposed scheme for (A) the proton‐coupled electron transfer of ARS (B) the oxidation and reduction of potassium hexacyanoferrate (II)/(III).

While energy efficiency is one variable to optimize, it is also important to consider the hazardous nature of the sorbents, coatings, electroactive molecules, solvents, and electrolytes used for conventional and/or electrochemical DAC; many of these materials represent a work, health, and safety risk. In search of an environmentally benign redox‐active molecule for aqueous electrochemical DAC, we employed Alizarin Red S (sodium 3,4‐dihydroxy‐9,10‐dioxo‐9,10‐dihydroanthracene‐2‐sulfonate), a water‐soluble anthraquinone dye, as the catholyte, 3‐pyridinecarboxamide (nicotinamide) as a hydrotropic agent, and potassium hexacyanoferrate (II) trihydrate as the anolyte (Scheme 1). Alizarin can be extracted from the dried roots of the madder plant, which grows in the wild as a weed and has long been consumed for medicinal purposes and as a natural food coloring.^[13]^

This work represents the first demonstration of electrochemical CO_2_ capture with Alizarin Red S (ARS). Additionally, while the potassium ferro/ferricyanide redox couple is commonly employed in aqueous redox flow batteries due to its reversibility and solubility, it is yet to be used for electrochemical DAC.^[14]^ In combination, this work represents a novel, green approach for electrochemical DAC. Overall, ARS was selected for the ability to achieve a high coulombic efficiency and capacity retention, its relatively non‐hazardous nature, and its low cost, which may cumulatively facilitate commercially viable DAC.

A range of organic molecules such as quinones,[^11^, ^15^] pyridines,[^16^, ^17^] and diazines[^17^, ^18^] have been characterized for electrochemical CO_2_ capture. The mechanism for capture varies depending on the availability of free protons. In an aprotic solvent system, the organic molecule is typically electrochemically reduced at the cathode to form an anion.[^7^, ^11^, ^17^] These negatively charged species are nucleophilic, so in the presence of CO_2_, the anions attack the δ+ partial charge to form a carbonate adduct – leaving behind other gaseous components found in ambient air, such as nitrogen and oxygen. Subsequently, the organic molecule can be electrochemically oxidized back to its neutral form, which releases a concentrated stream of gaseous CO_2_ that can be evacuated from the solution.

The mechanism for CO_2_ capture differs in a protic solvent system, such as water, because the electrochemically generated anions protonate before they react with CO_2_ molecules,^[18]^ leading to proton‐coupled electron transfer (PCET).^[18]^ Here, protonation of the anion species lowers the concentration of H^+^ ions relative to OH^−^ ions in solution, which increases the pH of water. When CO_2_ is then bubbled through solution, instead of directly reacting with the protonated organic molecule, CO_2_ reacts with the conjugate base OH^−^ ions to form HCO_3_^−^ ions. This functions similarly to enhanced ocean alkalinity concepts for atmospheric carbon dioxide removal, because more alkaline solutions can support a greater amount of dissolved inorganic carbon (DIC) as carbonate and bicarbonate ions.^[19]^ When the protonated organic molecule is electrochemically oxidized back to its neutral form, protons are lost from the organic molecule, which lowers the water′s pH and the water′s DIC capacity – yielding H_2_O and the outgassing of CO_2_ for sequestration.^[19]^

In previous work, we examined the electrochemical reversibility of archetypal organic molecules – pyrazine, 4,4′‐bipyridine, phenazine, 1,4‐benzoquinone, and 2,3,5,6‐tetrachloro‐p‐benzoquinone – for a non‐aqueous redox flow DAC process.^[17]^ This work demonstrated that phenazine was the most electrochemically reversible of the molecules examined. However, when considering the commercial deployment of a DAC system utilizing phenazine, its near insolubility in water, and poor solubility in commonly used aprotic solvents, such as acetonitrile (MeCN) and dimethyl sulfoxide (DSMO), hinders its suitability for scale‐up. Additionally, for environmental and safety reasons, aqueous redox flow systems are preferable to non‐aqueous systems because they avoid the consumption of large volumes of costly and often toxic organic solvents and supporting electrolytes. Lastly, due to the humidity of the ambient atmosphere, it is likely that purging air through an aprotic solvent will lead to the accumulation of water in the system, which could cause issues if the system is not designed to undergo a pH swing. Overall, we find it desirable to design an aqueous DAC process.

Relative to the equivalent materials and solvents used for a non‐aqueous process, those used in the present work offer substantial environmental, safety, and cost advantages. The catholyte, anolyte, solvent, and electrolyte employed in this work are classified as non‐hazardous materials by the Globally Harmonized System of Classification and Labelling of Chemicals (GHS), and the hydrotropic agent is characterized as a Category 2 A eye irritant.[^20^, ^21^, ^22^]

## Results and Discussion

Cyclic voltammetry measurements of ARS were first performed in an aqueous electrolyte solution purged with argon and later purged with CO_2_ to understand the influence of CO_2_ on redox behavior. Since potassium ferro/ferricyanide does not directly participate in the CO_2_ capture reaction beyond its role as an electron donor/accepter, cyclic voltammograms were only measured in an aqueous electrolyte solution purged with argon.

**Figure 1:**
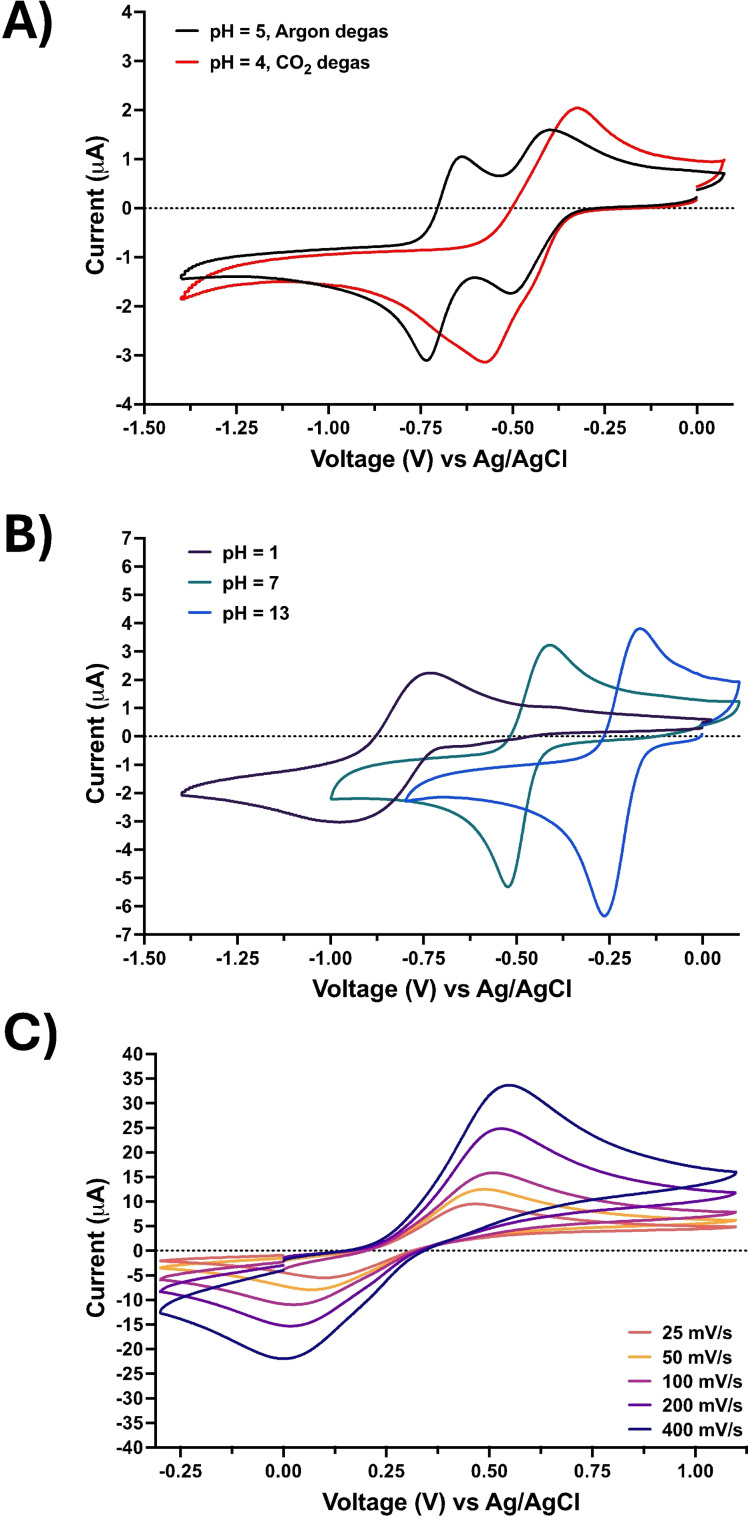
The cyclic voltammetry performed with a glassy carbon working electrode, Ag/AgCl reference electrode, and a platinum counter electrode of (A) ARS in a 0.1 M KCl in H_2_O solution purged with argon and CO_2_ at 100 mV/s scan rate; (B) pH 1 achieved with HCl dropped into a 0.1 M KCl in H_2_O solution, pH 7 achieved with a potassium dihydrate orthophosphate buffer in a 0.1 M KCl in H_2_O solution, and pH 13 achieved with a 0.1 M KOH in H_2_O solution – all solutions purged with argon and measured at 100 mV/s (C) potassium hexacyanoferrate (II)/(III) in a 0.1 M KCl in H_2_O solution with scan rates from 25 to 400 mV/s.

Notably, the voltammetry of ARS is strongly pH dependent. In the case of Figure 1A, when ARS is dissolved in an aqueous electrolyte solution and purged with argon, the pH was measured to be 5, and the ARS exhibited a two‐electron reduction and oxidation that occur in two separate steps. Conversely, when the same solution is subsequently purged with CO_2_, the pH decreases to 4, and the two‐electron redox processes occur in one single step.

To further explore the pH dependent voltammetry, the pH was controlled and tested, as seen in Figure 1B. As the pH of the aqueous electrolyte solution is lowered from pH 13 to 7 to 1, the half‐wave potential (E_1/2_) shifts anodically toward 0 V. Overall, the cyclic voltammograms align with the work of Turcanu and Bechtold.^[23]^ As demonstrated in Figure S2, both potassium hexacyanoferrate and ARS exhibit diffusion‐controlled behavior by demonstrating a linear relationship between the measured peak current and the square root of scan rate.

In an electrochemical CO_2_ capture process, the minimum theoretical energy required to capture CO_2_ is calculated from the half‐wave potential exhibited in argon and in CO_2_. As demonstrated by Barlow and Yang^[11]^ and ourselves,^[17]^ thermodynamic minima can be calculated using Gibbs Free Energy. In the present work, the E_1/2_ of ARS in argon is −0.696 V and the E_1/2_ in CO_2_ is −0.441 V, which yields a theoretical minimum energy of 24.6 kJe/mole of CO_2_. This thermodynamic minimum aligns with comparable systems; for instance, Seo and Hatton^[18]^ report a minimum of 35 kJe/mole of CO_2_ for their neutral red pH swing electrochemical DAC system, Jin *et al*.^[24]^ report 61.9 kJe/mole of CO_2_ for their pH swing electrochemical CO_2_ capture process, and Barlow and Yang^[11]^ report 34.7 kJe/mole of CO_2_ for their non‐aqueous tetrachloro‐p‐benzoquinone electrochemical process. Calculations for the thermodynamic minimum energy can be found in Supporting Information.

In testing of the electrochemical DAC system, CO_2_ capture measurements were performed using compressed air with a concentration of 400 ppm±12 ppm. During the experiments, a flow meter constantly supplied 20 mL/min of air, which was bubbled through a syringe to the ARS catholyte solution, and the exit stream was fed to a non‐dispersive infrared (NDIR) CO_2_, relative humidity, and temperature sensor. Pictures of the experimental setup can be found in Figure S5. For each sorption cycle, constant current constant voltage (CCCV) charging was employed. So, −31.25 mA of constant current was applied with a voltage cutoff of −1.75 V, at which point the voltage was held constant until current decreased to −10 mA or until a charging capacity of 46.9 mAh was reached. Then the system underwent a 4 minute rest cycle prior to the desorption process. During desorption, a constant current of 31.25 mA was applied with a voltage cutoff of 1.75 V, and a charging limit of 46.9 mAh.

**Figure 2:**
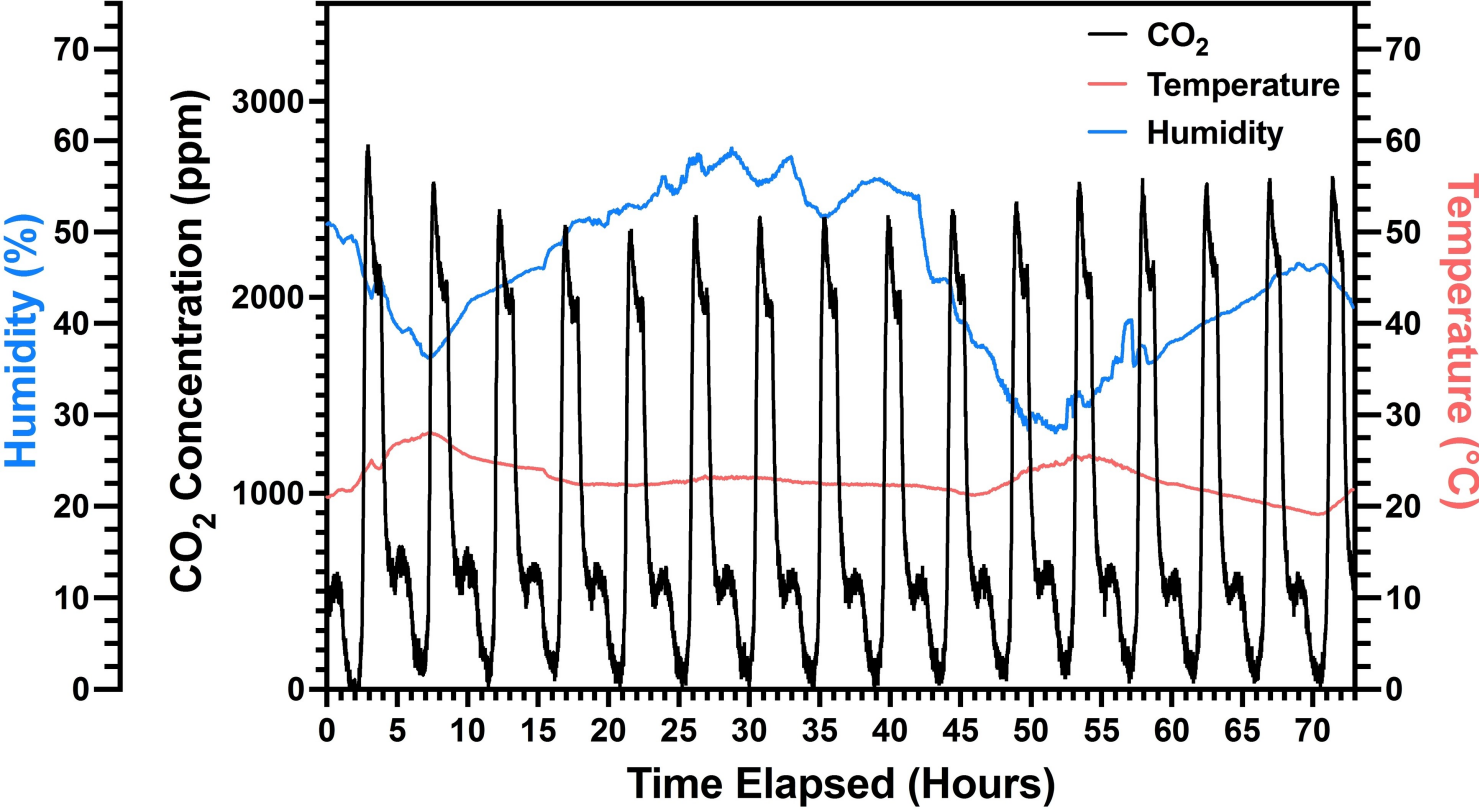
CO_2_ concentration, temperature, and relative humidity for the exit stream of air purged through the catholyte solution at a flow rate of 20 mL/min. CO_2_ capture performed using 50 mL of 20 mM ARS, 1 M Nicotinamide, 1 M KCl aqueous catholyte and 50 mL of 40 mM K_4_[Fe(CN)_6_], 1 M KCl aqueous anolyte.

**Figure 3:**
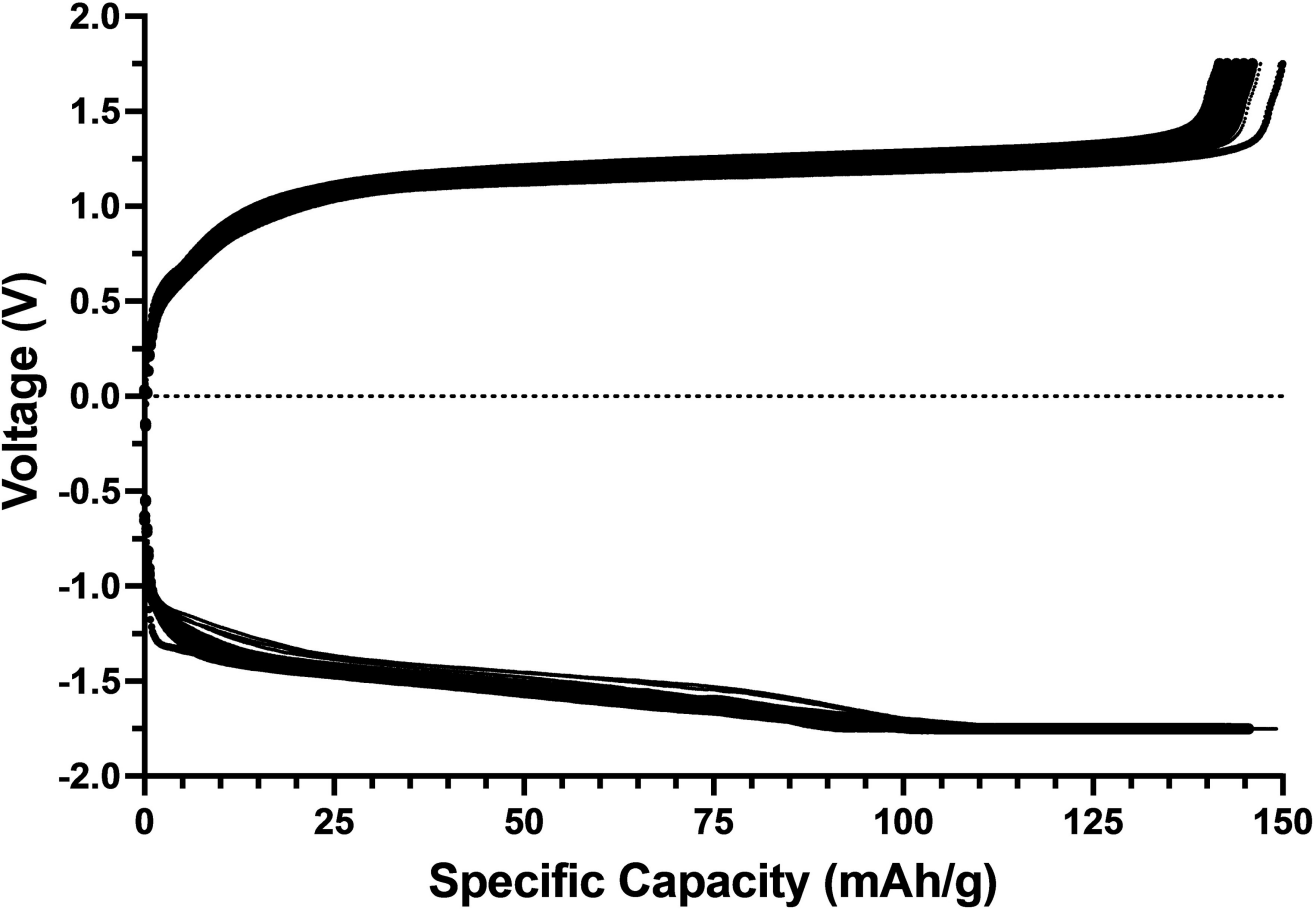
Charge/Discharge curves for the 16 cycles (72 hours) electrochemical DAC experiments performed with 50 mL of 20 mM ARS, 1 M Nicotinamide, 1 M KCl aqueous catholyte and 50 mL of 40 mM K_4_[Fe(CN)_6_], 1 M KCl aqueous anolyte.

The DAC absorption and desorption cycling results are shown in Figure 2 along with the humidity and temperature conditions. Critically, due to the nature of the CO_2_ capture mechanism, the system can operate in very humid conditions, which is a considerable hurdle for many temperature‐swing DAC systems.^[25]^ As seen in Figure 2, the capture and release of CO_2_ was highly repeatable over more than 72 hours of continuous testing. During these CO_2_ capture measurements, the system averaged a coulombic efficiency of 100 % and an average capacity retention of 99.6 %. Coulombic efficiency was calculated for each cycle based on the amount of charge passed during the charge cycle (sorption) divided by the amount of charged passed during the discharge cycle (desorption).^[26]^ Alternatively, capacity retention is defined as the amount of charge passed on the discharge cycle of cycle n relative to the amount of charge passed on the discharge cycle of n+1.^[26]^ From the charge/discharge voltage curves in Figure 3, it is evident that, much like the CO_2_ capture experiments, the charging and discharging of the system are highly repeatable without much deviation. To demonstrate even better consistency during CO_2_ capture/release, it would be advantageous to increase the rest time prior to sorption after the completion of desorption.

**Figure 4:**
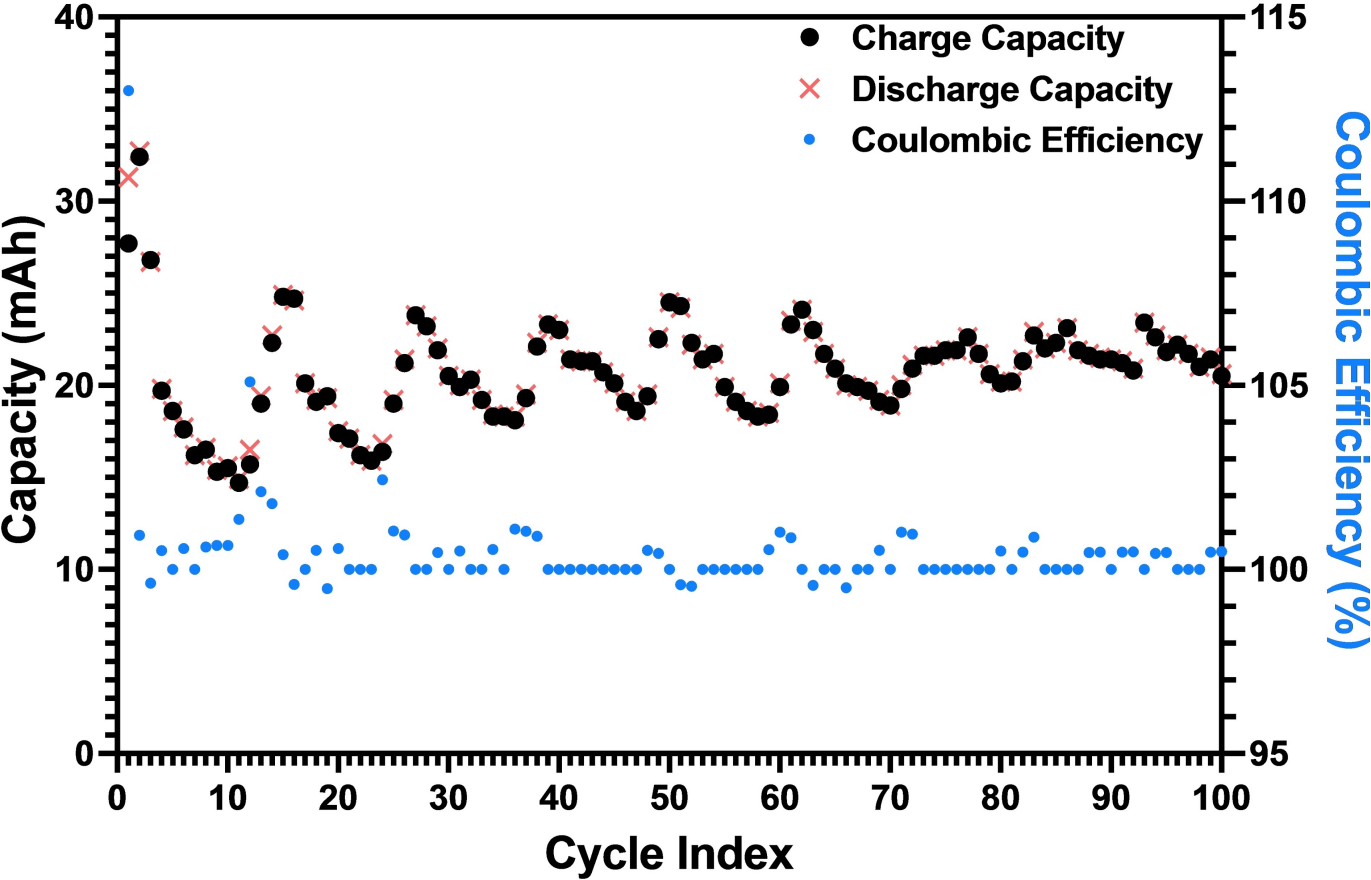
Charge/discharge capacity and coulombic efficiency through 100 cycles (205 hours) of continuous testing with 50 mL of 20 mM ARS, 1 M Nicotinamide, 1 M KCl aqueous catholyte and 50 mL of 40 mM K_4_[Fe(CN)_6_], 1 M KCl aqueous anolyte.

Given the high coulombic efficiency and capacity retention metrics exhibited during CO_2_ capture testing, we attempted to replicate the experiment in longer term electrochemical cycling measurements, as shown in Figure 4. In these experiments, the cell was charged with −25 mA of constant current and a −1.75 V cutoff, and then discharged with 25 mA of constant current and a 1.75 V cutoff – neither experiment had a set charging limit.

Testing revealed that pre‐and‐post sorption rest time strongly influenced both coulombic efficiency and capacity retention. When pre‐sorption rest times were too short – below approximately 20 minutes – capacity retention suffered; when post‐sorption rest times were too short or too long, coulombic efficiency suffered. By optimizing these factors, we were able to replicate the initial CO_2_ capture results and achieve an average coulombic efficiency of 100 % with an average capacity retention of 99.8 % over 100 cycles – operating continuously for more than 205 hours.

Notably, charge capacity varied between cycles depending on the ambient temperature in the laboratory – the local maxima coinciding with the warmer parts of the day, and the local minima coinciding with the cooler temperatures overnight. Additionally, as the temperature warmed up during the week, the capacity remained higher than during the cooler start of the week. This aligns with experimental work on redox flow batteries, which demonstrated that elevated temperatures can increase ionic conductivity and ionic mobility of the active and supporting electrolytes, thus increasing capacity relative to colder cycles.^[27]^

Overall, this work represents one of the more comprehensive demonstrations of continuous electrochemical DAC in the literature; Seo and Hatton^[18]^ reported 96 hours of continuous testing on neutral red, Zhu *et al*.^[28]^ demonstrated 72 hours of continuous testing on an electrochemical carbonate‐based system, and Li *et al*.^[29]^ performed approximately 264 hours of continuous testing on a hybrid electrochemical/thermal DAC system. Additionally, the coulombic efficiency reported in this work exceeds that of comparable work; Voskian and Hatton reported a coulombic efficiency of 90 %, Zhu *et al*. reported a coulombic efficiency of 90 %, Liu *et al*. reported a coulombic efficiency of 95.5 %, and Rahimi *et al*. reported coulombic efficiencies ranging from 96–100 % over five cycles.[^15^, ^28^, ^30^, ^31^]

### Flow Cell Optimization

In addition to reporting a non‐aqueous chemistry in our previous work,^[17]^ we also reported the design and development of a zero‐gap redox flow cell, which was 3D printed from polypropylene. To analyze the scale‐up of electrochemical DAC, the present work utilizes a flow cell with several improvements compared to the previous version regarding solvent leakage and flow field design.^[17]^

In the latest generation of our 3D printed flow cell, the nozzle flow factor was increased from 100 % to 105 % to intentionally produce slightly over‐extruded layers. Functionally, the flow factor is an extrusion multiplier, and a higher flow factor prevents the formation of small inter‐layer gaps as the print is generated. This printing variable was examined by O′Connor *et al*.^[32]^ who found that increasing a nozzle′s flow factor to 105 % reduced the dimensional accuracy of 3D printed flow cells but also lowered internal porosity and leakage. Small gaps along layer lines enable channeling of the solvent as it looks for the path of least resistance when traveling through the flow cell. For a device that must be well sealed to pressurized liquid and gas, it is optimal to over‐extrude the print in exchange for proper sealing. For this work, Verbatim polypropylene filament was selected over Fiberlogy because the filament provided a better seal at equivalent flow rates. Additionally, like O′Connor *et al*.,^[32]^ we used 100 % infill to provide compressive strength and minimize leakage.

It was critical to print the flow cell with polypropylene due to its strong chemical resistance relative to more common filament types such as polylactic acid (PLA), acrylonitrile butadiene styrene (ABS), and polyethylene terephthalate glycol (PETG). Using polypropylene, the flow cell can operate in a range of solvents including MeCN, DMSO, H_2_SO_4_/H_2_O, KOH/H_2_O, dimethyl formamide (DMF), and other commonly used solvents for electrochemistry.[^33^, ^34^]

Another critical performance aspect for the flow cell is internal resistance. In our testing, the Fumasep FAA‐3‐PK‐75 cation exchange membrane was selected for its low internal resistance and structural stability. The membrane is structurally reinforced with polyether ether ketone (PEEK), which is even more chemically resistant than polypropylene.^[35]^

**Figure 5:**
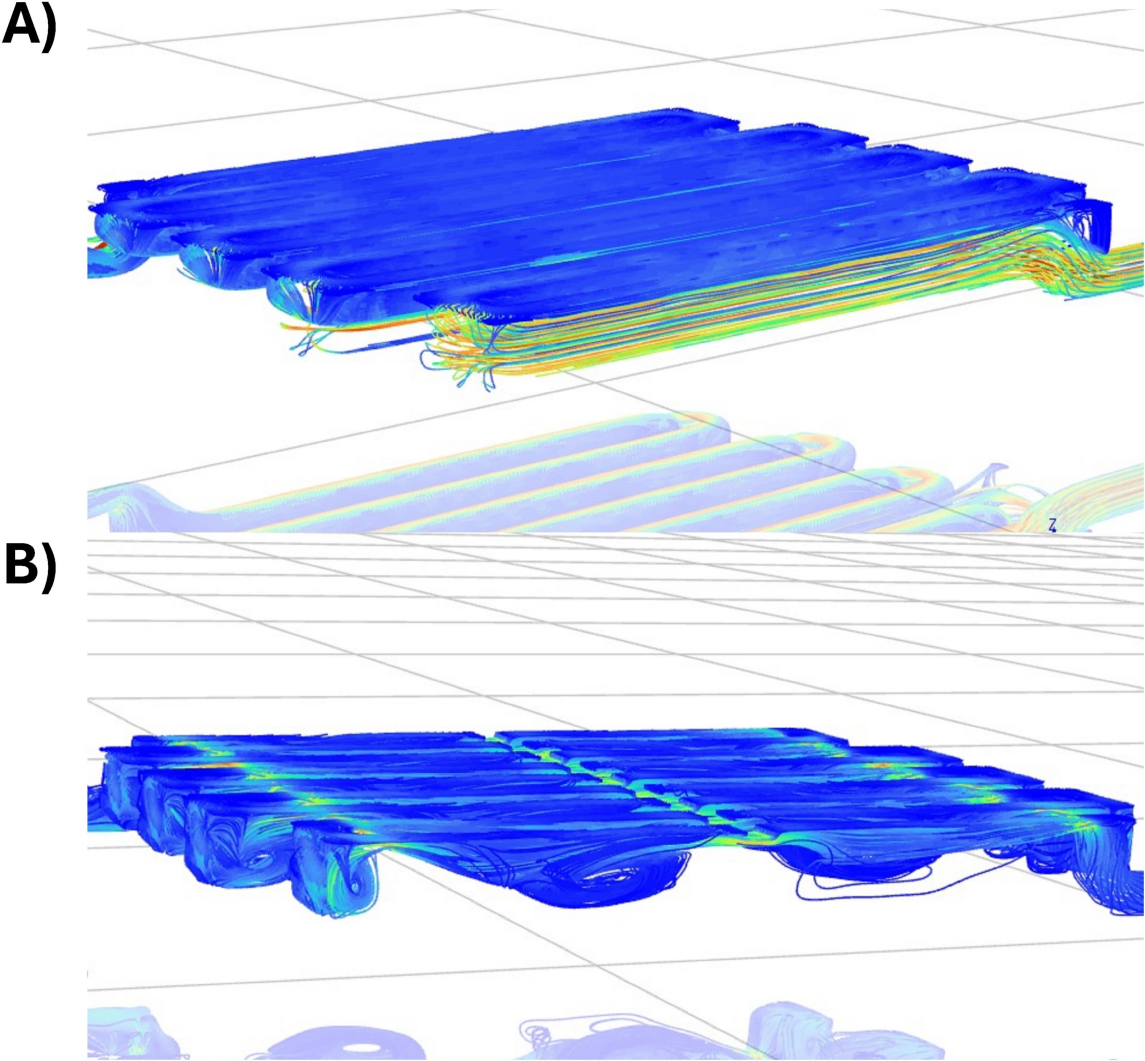
CFD simulations of water flowing through (A) the unmodified flow field (B) the modified flow field with agitators.

To further improve the performance of the flow cell, computational fluid dynamics (CFD) simulations were performed using Ansys Fluent software.^[36]^ Specifically, the geometry of the flow field was modified to iteratively enhance the interaction between the solution and the graphite felt electrode, which sits on top of the flow field. Initial CFD simulations of the unmodified flow field (Figure 5A) showed that the solution′s velocity vectors linearly followed the path of the flow field with minimal mixing along the z‐axis. The most turbulent portions of the unmodified flow field were at the end of each linear section (Figure 5A).

To improve mixing of the solution, flow agitators and constrictors in the shape of pyramids and rectangles were added to the flow field and simulated to understand their influence on flow velocity vectors. Through several rounds of simulation and design modification, it was found that the addition of long triangular extrusions on the edge of the flow field with a middle agitator could stimulate turbulent mixing zones. In these areas, solution would flow up or down the triangular ramp, hit the middle agitator and cause circular solution mixing (Figure 5B). Overall, this enhances the solution′s mixing and promotes movement of the solution to the electrode′s surface.

In the previous redox flow cell setup, a platinum foil current collector was used to facilitate electron transport from the power source to the graphite felt electrodes and back. The platinum current collectors maintained excellent conductivity, were electrochemically inert over the applied potentials, and were thin enough to enable a seal across the O‐ring. However, in trying to design a system that is both low‐cost and scalable, it became necessary to seek a replacement foil. Aluminum foil, although being affordable and perhaps the most widely available foil, degraded upon exposure to the applied electrical potentials. Brass and copper current collectors were also tested, and both suffered from corrosion and dissolution. Lastly, we tested a titanium foil, which was affordable, offered low Ohmic resistance, and was chemically inert over the applied voltages. As a result, we switched from platinum to titanium foil, which significantly lowered the cost of producing a fully assembled flow cell.

### Techno‐Economic Analysis

The deployment and commercialization of DAC relies heavily on lowering the energy consumption and cost per tonne of CO_2_ (tCO_2_) captured by such systems. When evaluating DAC systems, techno‐economic analyses are an important tool to identify primary cost drivers and to gain insight on the most impactful ways to drive future cost reductions.^[37]^ For an electrochemical DAC system, a techno‐economic analysis must calculate the present value of the costs accumulated throughout the system′s lifetime; this includes the cost of flow cells, peristaltic pumps, supporting electrolyte, catholyte, anolyte, solvent, maintenance, and electricity.

In our previous work, we developed a 3D printed redox flow cell for a non‐aqueous electrochemical DAC system, which employed phenazine as the redox‐active capturing agent. While flow batteries do not face the same current density constraints as conventional batteries, solubility is still an important consideration when designing a redox flow DAC process. One concern with our previous non‐aqueous system pertains to the poor solubility of phenazine in commonly used non‐aqueous electrochemical solvents, such as acetonitrile (MeCN) and dimethyl sulfoxide (DMSO). When solubility of the active species is low, particularly in a non‐aqueous process, large volumes of solvent and supporting electrolyte are required to capture large quantities of CO_2_. As a result, this could dramatically increase the accumulated costs of raw materials.

**Figure 6:**
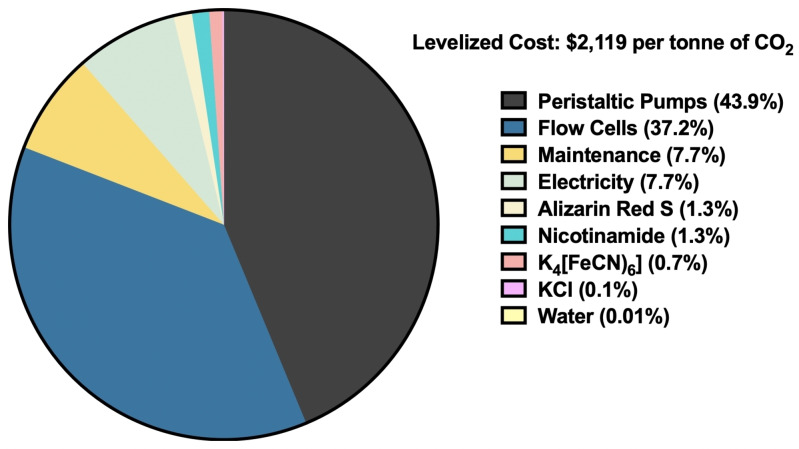
Breakdown of the levelized cost constituents for electrochemical DAC per tonne of CO_2_.

To assess the techno‐economic viability of an ARS/K_4_[(FeCN)_6_] DAC process, we performed a discounted cash flow for the 10 year operation of a demonstration‐scale version of our DAC process – accounting for maintenance and periodic full replacement of the solvent system, fuel cells, and pumps (Figure 6). If the system used for laboratory testing, with a current density of 2.5 mA/cm^2^ and an electrode surface area of 9.07 cm^2^, were to be used for tonne‐scale CO_2_ removal, this would translate to a LCOC of more than US$84,000 per tCO_2_ – rendering the process economically unviable. However, by simply increasing current density to 25 mA/cm^2^ and the size of each cell to 100 cm^2^, the levelized cost decreases to US$2,119 per tCO_2_. By further increasing current density to 100 mA/cm^2^ and the size of the working electrode to 100 cm^2^, the LCOC drops to $453 per tCO_2_. Advancements in current density can be achieved by using a thinner membrane, increasing the concentration of ARS and K_4_[(FeCN)_6_], and further optimizing the flow cell design. In this work, we utilized a 20 mM ARS catholyte solution to limit cycle times during testing, but with a higher hydrotropic agent concentration, it is possible to increase the concentration of ARS and therefore current density as well.

A breakdown of accumulated costs over the course of 10 years of operation shows that capital costs for fuel cells and peristaltic pumps comprise over 81 % of the LCOC. Using a moderately sized flow cell with modest current density requires many flow cells and pumps to electrolyze the catholyte/anolyte solutions, which is why current density and electrode surface area have a significant influence on levelized costs. Additionally, because annual maintenance comprises 3 % of the installed equipment costs, and equipment dominates the LCOC, maintenance is responsible for over 7 % of accumulated costs. Electricity is the last main cost contributor, as it is the energy source for the process, but it is likely less of a cost factor for an electrochemical DAC process than for a conventional temperature/pressure swing system.

**Figure 7:**
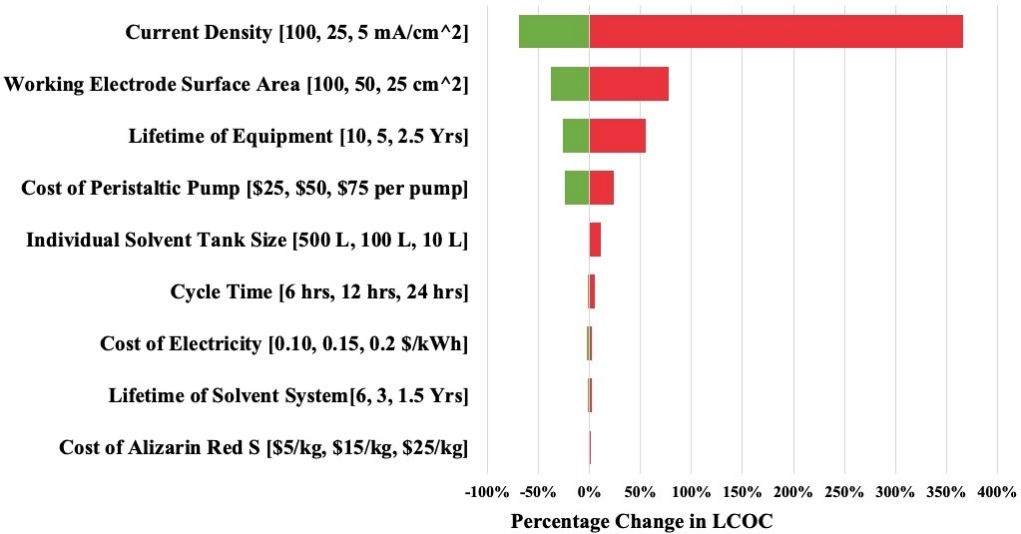
Tornado analysis of the most influential cost factors.

As seen in Figure 7, we performed a sensitivity analysis of 9 key factors to determine their uncertainty and influence on the LCOC. To calculate the influence of an individual variable, all parameters except for the variable of interest were held constant. Only four variables were found to have a strong influence on the LCOM: current density, working electrode surface area, equipment lifetime, and the cost of peristaltic pumps. Overall, current density is the most influential parameter because it determines the number of flow cells and pumps required to electrolyze the solution. With greater current density and larger electrodes, fewer cells and pumps are required to achieve the same output, which is why the LCOC can be severely reduced by modifying these variables. For the same reason, the surface area of the working electrode has a strong influence on the LCOC. Taken together, improving current density and producing a larger flow cell collectively serve as future priorities to unlock low‐cost Direct Air Capture. The equipment lifetime of the cells and pumps naturally also exert a strong influence on the LCOC due to the share of costs that these two factors comprise; short equipment lifetimes only exacerbate the share of costs comprised by pumps and flow cells. Lastly, since each flow cell requires a peristaltic pump, the cost of pumps scales linearly with the number of flow cells. As a result, the capital cost of each peristaltic pump has strong influence on the LCOC. The discounted cash flow can be found in the supporting information.

Performing a techno‐economic analysis of the electrochemical DAC system highlights the importance of utilizing low‐cost redox flow equipment. While flow cells account for 37.2 % of accumulated costs in our model, if a more expensive conventional flow cell were purchased and used, the levelized cost of CO_2_ would dramatically increase. In contrast to conventional cells, which cost several thousand dollars each, the assembled redox flow cells developed for this work, which includes 3D printing filament, O‐rings, aluminum endplates, nuts and bolts, tubing, hose clamps, electrodes, a membrane, and current collectors, cumulatively cost approximately US $42 per flow cell. Approximately 100 g, or US$7.5, of polypropylene filament is used to print the cell itself. However, if cells were purchased commercially, a similarly sized cell would cost at least US$2,000 and more typically would cost over US$3,500. If the flow cell were to cost US$2,000, this increases the LCOC from US$2,119 per tCO_2_ to over US$42,000 per tCO_2_, which underscores the importance of using low‐cost equipment.

## Conclusions

In this paper, we characterized the electrochemical behavior of a green redox flow DAC process using an aqueous electrolyte solution. Utilizing ARS for an aqueous electrochemical DAC process offers environmental, health, and safety benefits, and the combined use of a 3D printed flow cell offers cost reductions. Our work illustrated that ARS is a reversible organic catholyte material with the potential to lower energy consumption to a thermodynamic minimum of 24.6 kJe/mol of CO_2_ captured. Through CO_2_ capture measurements, we were able to demonstrate reversible sorption and desorption for more than 72 hours. The results were then replicated through 100 cycles (205 hours) of electrochemical cycling tests, which averaged a coulombic efficiency of 100 % and a capacity retention of 99.8 %. In addition to the chemistry, we explained the design and development process for a 3D printed redox flow cell, and we performed CFD simulations to improve the flow of solution through the device. Lastly, we performed a techno‐economic analysis of the reported electrochemical DAC system to identify major cost contributors and opportunities to reduce costs to below US$454 per tCO_2_. Overall, this examination of an aqueous electrochemical DAC system represents a step toward low‐cost, scalable, reversible Direct Air Capture.

## Conflict of Interests

The authors declare no conflict of interest.

1

## Supporting information

As a service to our authors and readers, this journal provides supporting information supplied by the authors. Such materials are peer reviewed and may be re‐organized for online delivery, but are not copy‐edited or typeset. Technical support issues arising from supporting information (other than missing files) should be addressed to the authors.

Supporting Information

## Data Availability

The data that support the findings of this study are available in the supplementary material of this article.
