# Supplementary material for: Aqueous Electrochemical Direct Air Capture Using Alizarin Red S
Source: ChemSusChem. 2024 Oct 30;18(3):e202401315. doi: 10.1002/cssc.202401315 (PMC11789980; doi:10.1002/cssc.202401315)
Supplement: Supplementary file 1 — Supporting Information [file CSSC-18-e202401315-s001.pdf]

ChemSusChem

Supporting Information

Aqueous Electrochemical Direct Air Capture Using Alizarin Red S

Samuel R. Wenger* and Deanna M. D'Alessandro

Aqueous Electrochemical Direct Air Capture Using Alizarin Red S

Samuel R. Wenger [a, b] and Deanna M. D'Alessandro [a, b*]

[a] S. Wenger, Prof. D. D'Alessandro
School of Chemical and Biomolecular Engineering, Faculty of Engineering
The University of Sydney
Darlington, NSW, 2008, Australia
*Corresponding Author E-mail: deanna.dalessandro@sydney.edu.au

[b] S. Wenger, Prof. D. D'Alessandro
School of Chemistry, Faculty of Science
The University of Sydney
Camperdown, NSW, 2006, Australia

Materials and Methods

Materials

Alizarin Red S, potassium hexacyanoferrate (II) trihydrate and nicotinamide were purchased from Merck. Potassium Chloride was purchased from Ajax Finechem. Ethylene Propylene (EPDM) O-rings were purchased from Hydraulic Seals Solutions. CT GF020 Graphite Felt and Fumasep FKD-PK-75 membranes were purchased from Fuel Cell Store. Polypropylene 3D printing filament was sourced from Verbatim. The flow cell was printed with a Prusa Mini +. The 30% K33 CO_2 sensor was purchased from CO_2Meter. Compressed air was purchased from BOC, which is comprised of 78% N_2, 20.8% O_2, and approximately 0.04% CO_2.

METHODS:

Cyclic Voltammetry (CV). CVs were obtained using an EC Epsilon potentiostat/galvanostat in a glass electrochemical cell with 0.1 M KCl in H_2O electrolyte solution, 1 mm diameter glassy carbon working electrode, an Ag/AgCl reference electrode, a platinum counter electrode. The electrolyte solution was purged with Argon or CO_2 for 10 minutes prior to the measurement depending on which gaseous environment was being studied. Data were analyzed and plotted using GraphPad Prism.

Chronopotentiometery (CP) and CO_2 Capture Measurements. Chronopotentiometry measurements were performed using Neware Battery Tester in a 3D printed flow cell with 50 mL of either 20 mM Alizarin Red S, 1 M Nicotinamide, and 1 M KCl in deionized water or 40 mM $K_4[(FeCN)_6]$ and 1 KCl in deionized water. CO_2 capture measurements were performed with compressed air to simulate Direct Air Capture conditions. Data were analyzed and plotted using GraphPad Prism software package.

For cycling, 25 mA of current was applied during the absorption process, and -25 mA was applied during the desorption process. Absorption or desorption occurred for 60 minutes or until a voltage cutoff of 1.3 V or -1.3 V was reached.

To calculate coulombic efficiency, the charge passed during each charge and discharge cycle were calculated by multiplying current and the time of each cycle. Then for the nth cycle, the charge passed during desorption segments was divided by the charge passed for each absorption cycle.

CO_2 capture was measured with a 033-9-0010 K33 BLG 30% CO_2 + RH/T Data Logging Sensor purchased from CO_2Meter. The sensor has a reported accuracy of ± 0.2% vol CO_2 or ± 3% of the measured value.

Experimental Setup. A round bottom flask (RBF) containing 50 mL of the catholyte and a separate RBF containing 50 mL of the anolyte were connected to a peristaltic pump with a flow rate of 10 mL/min. The solutions were pumped to the entry nozzles of their respective half-cell where the solutions flowed through the flow field, over the graphite felt electrode, which was connected to the potentiostat via a titanium foil current collector. After the solutions reacted at the electrode, they are pumped back to their respective RBF. In the catholyte RBF, compressed air is bubbled through the solution at a rate of 20 mL/min, and the exit concentration of CO_2 is monitored and logged by the NDIR CO_2 sensor.

3D Printing. CAD models were designed in Fusion 360. Models were sliced with Prusa Slicer. 3D printing was performed using 1.75 mm Fiberology Polypropylene filament. Redox flow cells were printed with a nozzle temperature of 240 °C, no bed temperature heating, a 0.4 mm diameter brass nozzle, and a 0.2 mm layer height. Supports were drawn using the circular brush paint-on-support tool.

CFD Simulations. Simulations were performed in Ansys Fluent software. The established inlet and outlet valves were the flow cell's nozzles, and a wall flush to the flow field was created. The selected fluid was water, and the flow was simulated with a velocity of 0.053 m/s. The simulation ran until all residuals were lower than $1e^{-6}$.

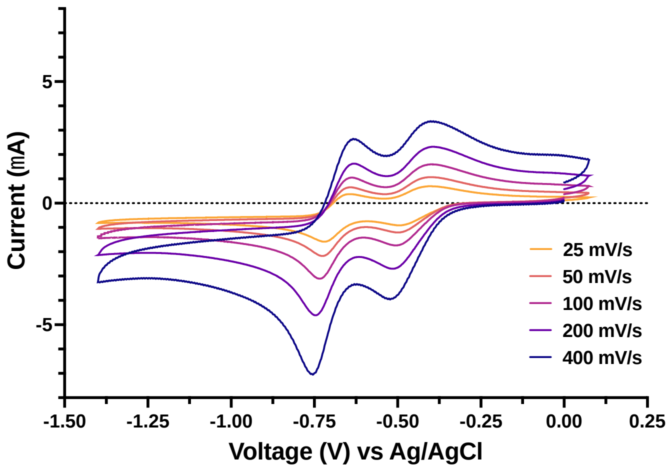

Figure S1: ARS in a 0.1 M KCl in H_2O solution purged with N_2 with scan rates from 25 to 400 mV/s.

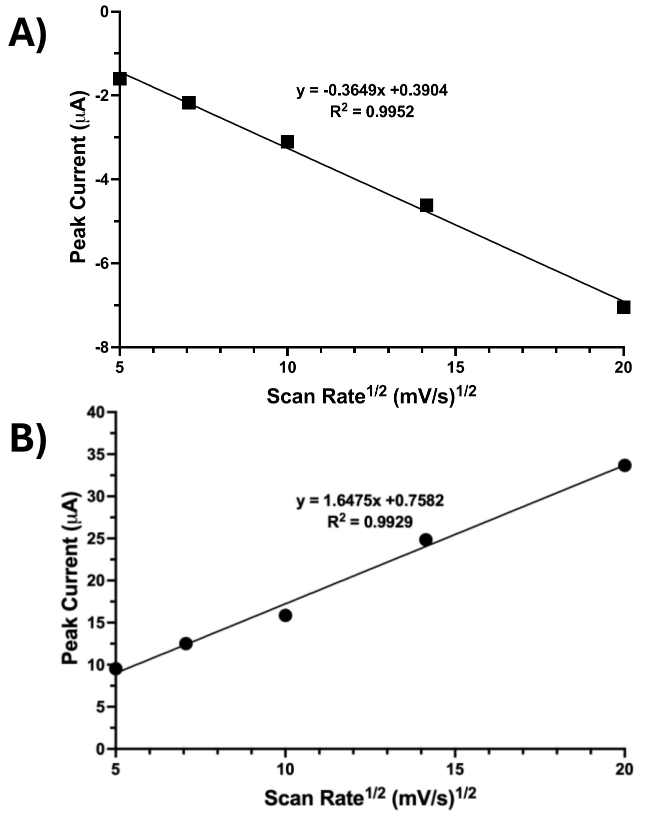

Figure S2: Scan rate dependence plot for (A) alizarin red s in a 0.1 M KCl in H_2O solution purged with N_2; (B) potassium hexacyanoferrate (II)/(III) in a 0.1 M KCl in H_2O solution purged with N_2.

As determined by the Randles-Sevcik equation, in a freely diffusing, reversible redox process, current should linearly scale with the square root of scan rate.[1] As seen with Figure S2A and S2B, both Alizarin Red S and potassium hexacyanoferrate (II) display strong linear relationships between peak current and the square root of scan rate with an R^2 value of 0.9952 and 0.9929, respectively. This suggests that both processes are freely diffusion and electrochemically reversible during cyclic voltammetry measurements. Peak current was calculated from the cathodic scan for alizarin red s and from the anodic scan for potassium ferrocyanide.

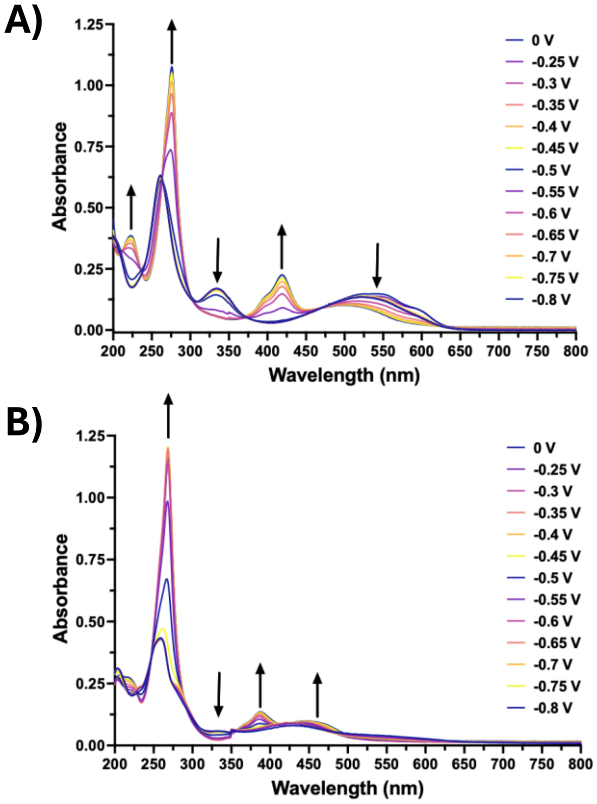

Figure S3: UV-Vis SEC of (A) Alizarin Red S in a 0.1 M KCl in H_2O solution purged with argon; (B) Alizarin Red S in a 0.1 M KCl in H_2O solution purged with CO_2

To further examine the interaction between ARS and CO_2, ultraviolet-visible (UV-Vis) absorption spectroelectrochemistry (SEC) was performed. In this experiment, UV-Vis SEC reveals changes in the absorption spectra as ARS that is electrochemically reduced in solutions purged with either argon or CO_2. As seen in Figure S3A, when ARS is electrochemically reduced in an aqueous electrolyte solution purged with argon, new absorption peaks form at 219 and 418 nm. Additionally, the initial peak at 258 nm increases in absorption and undergoes a slight shift to absorb at 276 nm. Lastly, the initial peak at 327 nm and the broad peak at approximately 530 nm decreases in absorption as the ARS is electrochemically reduced. In contrast, when the aqueous electrolyte solution is purged with CO_2 and electrochemically reduced in the same stepwise fashion, there is a relatively less dramatic decrease in absorption at 327 nm. Moreover, the peak that initially formed at 418 nm in the argon-purged solution shifts to 382 nm in the CO_2-purged solution, and the increase is more muted. Similarly, the broad peak that initially absorbed at 530 nm shifts to approximately 450 nm, and instead of decreasing absorption upon reduction, there appears to be an increase in absorption in the presence of CO_2.

While UV-Vis SEC of ARS has not been reported in the literature, Tong *et al*.[2] performed the measurement on 9,10-anthraquinone-2,7-disulfonic acid (AQDS), a sufficient analog. When the AQDS is electrochemically reduced in their UV-Vis SEC, the shift in spectra is attributed to the formation of the protonated hydroquinone form. In the behavior reported below for ARS, there is a very similar shift, which can be attributed to the formation of the electrochemically reduced and protonated form of ARS. The differences between Figure S3A and S3B can be attributed to the different protonation states caused by gaseous purging of the solutions.[3]

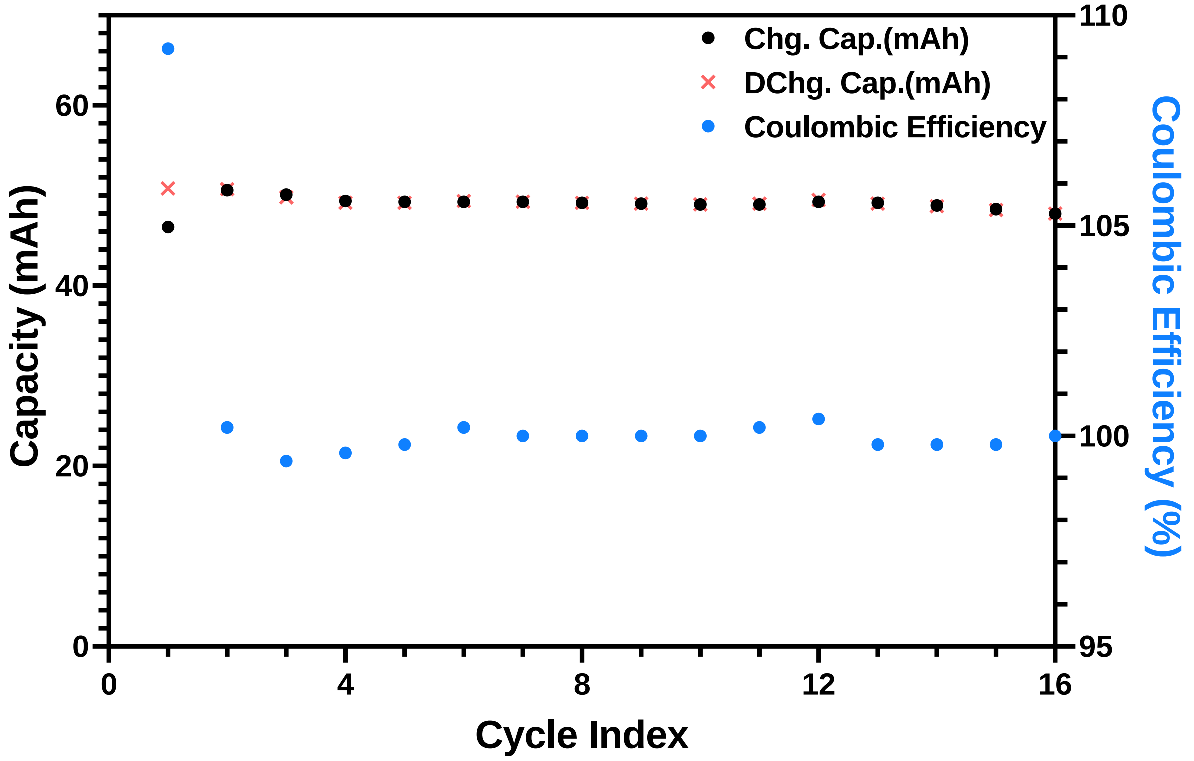

Figure S4: Charge/discharge capacity and coulombic efficiency through 16 cycles (72 hours) of continuous CO_2 capture testing with 50 mL of 20 mM ARS, 1 M Nicotinamide, 1 M KCl aqueous catholyte and 50 mL of 40 mM $K_4[Fe(CN)_6]$, 1 M KCl aqueous anolyte.

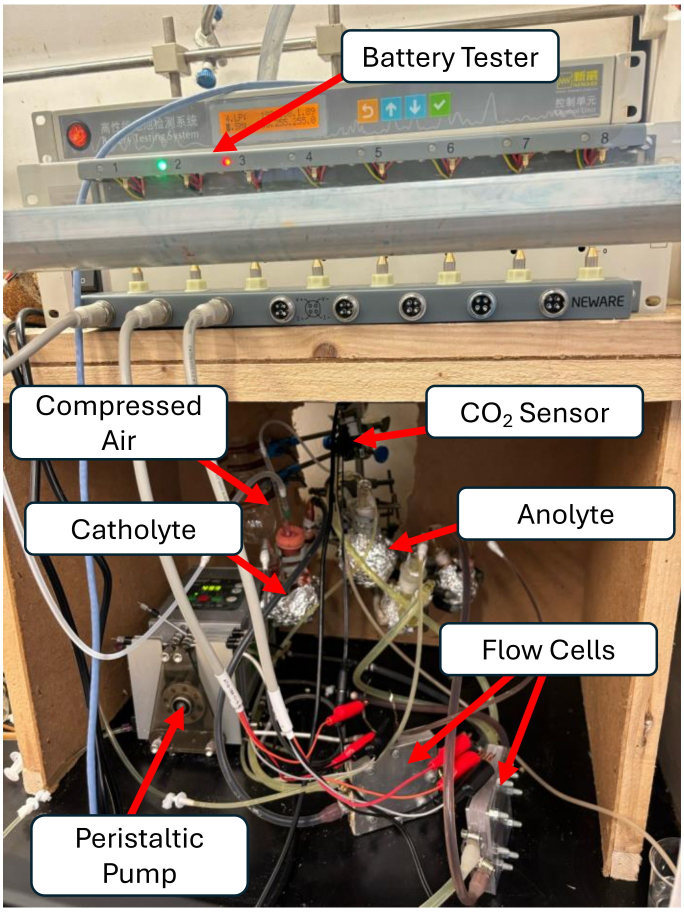

Figure S5: Photograph of the setup for electrochemical DAC experiments.

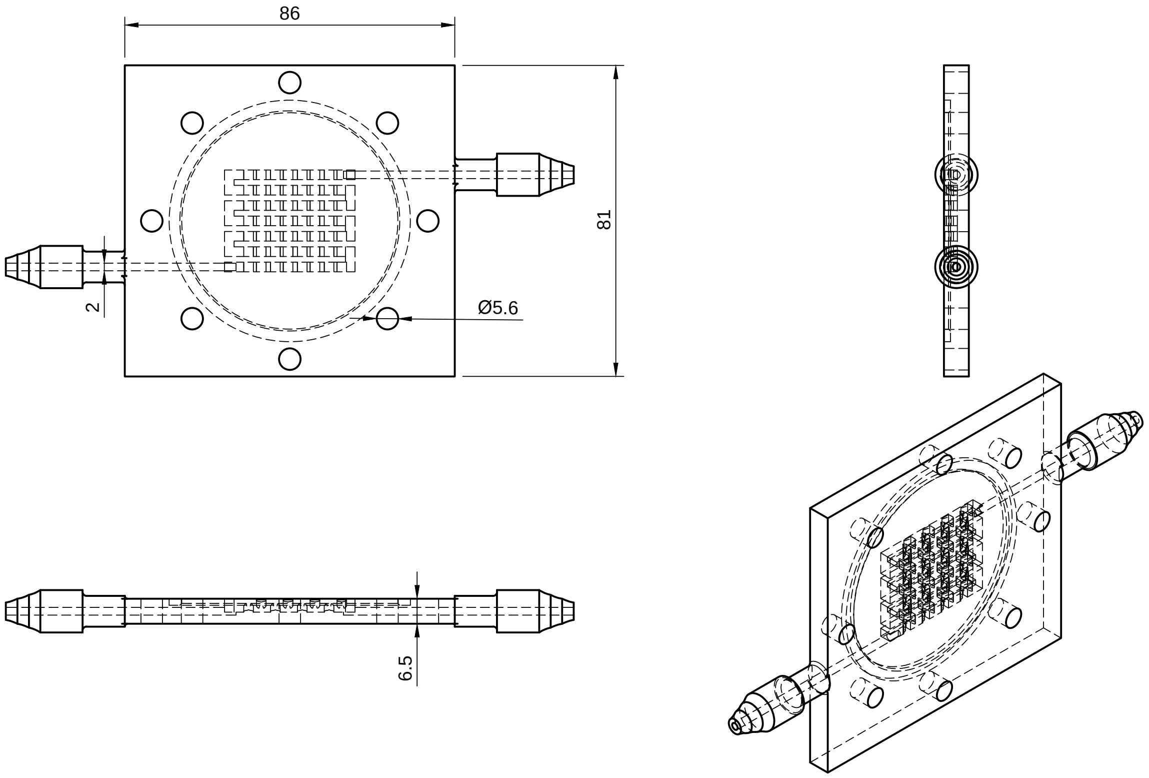

Figure S6: Schematic drawings for the 3D printed flow cell.

Sample Minimum Theoretical Energy Calculations

$\Delta G_{(CO2\_min)} = -mF\Delta E_{(1/2)}$

Where ΔG is Gibbs free energy for the electrochemical redox swing, m is the number of CO_2 molecules bound per electron passed, F is Faraday's constant, and ΔE is the difference in potentials between $E_{(1/2)}$ of CO_2 capture and release, as demonstrated by Barlow and Yang.[11]

Table S1: Theoretical Thermodynamic Minimum Calculations for Alizarin Red S

$E_{(100\%\ Argon)} = -0.696$ V
$E_{(100\%\ CO2)} = -0.441$ V
$\Delta E = -0.696 \text{ V} - -0.441 \text{ V} = -0.255$ V
e^- passed per CO_2 molecule = 1
$\Delta G_{(CO2\_min)} = -1*(96.485 \text{ kC/mol})*(-0.255 \text{ V}) = 24.6$ kJ/mol

Levelized Cost Calculations

To yield a levelized cost of CO_2 (LCOC), the present value of accumulated DAC costs must be divided by the present value of accumulated CO_2 production, as shown below.

Levelized Cost of CO_2 (LCOC) = $\frac{\sum_{t=0}^{N}\frac{C_t}{(1+r)^t}}{\sum_{t=0}^{N}\frac{P_t}{(1+r)^t}}$

LCOC can be calculated where C_t is the annual cost of production in year t, P_t is the number of tonnes of CO_2 captured in year t, r is the real cost of capital, and N is 10 years.

Year	0	1	2	3	4	5	6	7	8	9	10
Material 1: KCl		6.71	0.00	0.00	6.71	0.00	0.00	6.71	0.00	0.00	6.71
Material 2: Alizarin Red S		75.83	0.00	0.00	75.83	0.00	0.00	75.83	0.00	0.00	75.83
Material 3: Nicotinamide		72.06	0.00	0.00	72.06	0.00	0.00	72.06	0.00	0.00	72.06
Material 4: K4FeCN6		36.93	0.00	0.00	36.93	0.00	0.00	36.93	0.00	0.00	36.93
Solvent 1: Water		0.40	0.00	0.00	0.40	0.00	0.00	0.40	0.00	0.00	0.40
Assembled Fuel Cells	2671.07	0.00	0.00	0.00	0.00	2671.07	0.00	0.00	0.00	0.00	2671.07
Peristaltic Pumps	3150.00	0.00	0.00	0.00	0.00	3150.00	0.00	0.00	0.00	0.00	3150.00
Maintenance		174.63	174.63	174.63	174.63	174.63	174.63	174.63	174.63	174.63	174.63
Electricity		173.80	173.80	173.80	173.80	173.80	173.80	173.80	173.80	173.80	173.80
**Annual Total Cost**	5821.07	540.35	348.43	348.43	540.35	6169.50	348.43	540.35	348.43	348.43	6361.42
**PV of Annual Total Cost**	5821.07	505.96	305.50	286.06	415.39	4440.92	234.85	341.03	205.91	192.81	3296.09
**PV of Total Production Cost**	**16045.58**										
Yearly CO2 Capture	0	1	2	3	4	5	6	7	8	9	10
**Annual CO2 Capture Volume**		1.07	1.07	1.07	1.07	1.07	1.07	1.07	1.07	1.07	1.07
**PV of Annual CO2 Capture Volume**	0.00	1.00	0.94	0.88	0.82	0.77	0.72	0.67	0.63	0.59	0.55
**PV of CO2 Capture Volume**	7.57	tonnes									
**Unit Cost of CO2**	**2118.50**	**$/tonne**									

Table S2: Discounted Cash Flow for the Base Case TEA Scenario

Year	0	1	2	3	4	5	6	7	8	9	10
Material 1: KCl		6.71	0.00	0.00	6.71	0.00	0.00	6.71	0.00	0.00	6.71
Material 2: Alizarin Red S		75.83	0.00	0.00	75.83	0.00	0.00	75.83	0.00	0.00	75.83
Material 3: Nicotinamide		72.06	0.00	0.00	72.06	0.00	0.00	72.06	0.00	0.00	72.06
Material 4: K4FeCN6		36.93	0.00	0.00	36.93	0.00	0.00	36.93	0.00	0.00	36.93
Solvent 1: Water		0.40	0.00	0.00	0.40	0.00	0.00	0.40	0.00	0.00	0.40
Assembled Fuel Cells	462.10	0.00	0.00	0.00	0.00	462.10	0.00	0.00	0.00	0.00	462.10
Peristaltic Pumps	400.00	0.00	0.00	0.00	0.00	400.00	0.00	0.00	0.00	0.00	400.00
Maintenance		25.86	25.86	25.86	25.86	25.86	25.86	25.86	25.86	25.86	25.86
Electricity		109.45	109.45	109.45	109.45	109.45	109.45	109.45	109.45	109.45	109.45
**Annual Total Cost**	862.10	327.23	135.31	135.31	327.23	997.41	135.31	327.23	135.31	135.31	1189.33
**PV of Annual Total Cost**	862.10	306.41	118.64	111.09	251.56	717.96	91.20	206.52	79.96	74.88	616.24
**PV of Total Production Cost**	**3436.56**										
Yearly CO2 Capture	0	1	2	3	4	5	6	7	8	9	10
**Annual CO2 Capture Volume**		1.07	1.07	1.07	1.07	1.07	1.07	1.07	1.07	1.07	1.07
**PV of Annual CO2 Capture Volume**	0.00	1.00	0.94	0.88	0.82	0.77	0.72	0.67	0.63	0.59	0.55
**PV of CO2 Capture Volume**	7.57	tonnes									
**Unit Cost of CO2**	**453.73**	**$/tonne**									

Table S3: Discounted Cash Flow for the Optimistic Case TEA Scenario

References

[1] N. Elgrishi, K. J. Rountree, B. D. McCarthy, E. S. Rountree, T. T. Eisenhart, J. L. Dempsey, *J. Chem. Educ.* **2018**, *95*, 197–206.
[2] L. Tong, Q. Chen, A. A. Wong, R. Gómez-Bombarelli, A. Aspuru-Guzik, R. G. Gordon, M. J. Aziz, *Phys. Chem. Chem. Phys.* **2017**, *19*, 31684–31691.
[3] A. Turcanu, T. Bechtold, *Dyes and Pigments* **2011**, *91*, 324–331.
[4] J. M. Barlow, J. Y. Yang, *J. Am. Chem. Soc.* **2022**, *144*, 14161–14169.
